# Direct Discharge from the Critical Care Resuscitation Unit: Results from a Longitudinal Assessment

**DOI:** 10.1155/2023/2213185

**Published:** 2023-10-30

**Authors:** Quincy K. Tran, Austin Widjaja, Anya Plotnikova, Jerry Yang, Jacob Epstein, Alexa Aquino, Fernando Albelo, Taylor Kowansky, Isha Vashee, Samuel Austin, Daniel J. Haase, Emily Esposito

**Affiliations:** ^1^Research Associate Program in Emergency Medicine and Critical Care, Department of Emergency Medicine, University of Maryland School of Medicine, Baltimore, MD, USA; ^2^The R. Adams Cowley Shock Trauma Center, University of Maryland School of Medicine, Baltimore, MD, USA; ^3^Department of Emergency Medicine, University of Maryland School of Medicine, Baltimore, MD, USA

## Abstract

**Background:**

The critical care resuscitation unit (CCRU) facilitates interhospital transfer (IHT) of critically ill patients for immediate interventions. Due to these patients' acuity, it is uncommon for patients to be directly discharged home from this unit, but it does happen on occasion. Since there is no literature regarding outcomes of patients being discharged from a resuscitation unit, our study investigated these patients' outcome at greater than 12 months after being discharged directly from the CCRU.

**Methods:**

We performed a retrospective cohort study of all adult patients directly discharged from the CCRU between January 01, 2017, and December 31, 2020. The primary outcome was number of ED visits or hospitalizations within 6 months. Secondary outcomes were number of ED visits or hospitalizations within 6, 12, and >12 months from CCRU discharge.

**Results:**

We analyzed 145 patients' records. Mean age was 56 (standard deviation [SD] ± 19), with a majority being male (72%) and Caucasian (58%). The most common discharge destination was home (139 patients, 96% of total subjects) versus hospice (2%) or nursing facilities (2%). Most patients (55%) did not have any hospital revisits within the first 6 months of discharge, while 31% had 1-2 revisits, and 14% had ≥3 revisits. The most common discharge diagnoses were soft tissue infection (16.5%), aortic dissection (14%), and stroke (11%). Factors which were associated with a greater likelihood of any return hospital visit within 6 months receiving mechanical ventilation during CCRU stay (coefficient −2.23, 95% CI 0.01–0.87, *P*=0.036), while high hemoglobin on CCRU discharge was associated with no ED revisit (coeff. 0.42, 95% CI 1.15–2.06, *P*=0.004).

**Conclusions:**

Most patients who were discharged from the CCRU did not require any hospital revisits in the first 6 months. Requiring mechanical ventilation and having soft tissue infection were associated with high unplanned hospital revisits following discharge. Further research is needed to validate these findings.

## 1. Introduction

Directly discharging patients from an intensive care unit (ICU) has become a more familiar practice in hospitals in recent years [1, 2]. It has been postulated that this is a product of an increased burden of the need for ICU beds and decreased availability of hospital ward beds; however, this correlation has not previously been demonstrated [1]. The proposed benefits of directly discharging patients from the ICU include, but are not limited to, decreased hospital length of stay, increased availability of ICU beds, and decreased risks of adverse events associated with transfers of care.

Current literature on the effectiveness of direct discharges from the ICU is limited at this time, especially in the United States. However, existing studies have demonstrated a low mortality and low rate of hospital readmission in these patients, along with high patient and family satisfaction with the discharge process [1, 3–6]. Existing studies also showed that those who are directly discharged home are more often young and otherwise healthy, with few comorbidities. They often sustained disease processes, which involved a single-organ system and is reversible, that can be readily treated [1]. A barrier to directly discharging patients home from the ICU may be clinicians' comfort with the discharge process. This appears to be a more common practice amongst clinicians in the ICU with an emergency medicine background [3].

The Critical Care Resuscitation Unit (CCRU) at the University of Maryland Medical Center (UMMC) is a unique ICU that cares for both intrahospital direct admissions and interfacility transfers of critically ill patients with medical, surgical, or traumatic disease states [7]. Occasionally, patients are transferred to the CCRU without a clear diagnosis or in anticipation of the need for a particular surgical intervention. After careful history, physical examination, review of imaging, and consultation with the appropriate specialty service, it may be determined that operative intervention is not required [8]. Additionally, the clinical picture can improve while in the CCRU and if no operative intervention is planned, a certain population of patients may be stable for transfer to non-ICU level of care or even discharge from the UMMC to appropriate outpatient facilities. The CCRU is staffed 24 hours a day by intensivists who are residency trained in emergency medicine and fellowship trained in critical care medicine and have expertise and familiarity in managing severe disease processes.

Although it is uncommon, the CCRU does discharge a select population of patients to outpatient settings, without going through the traditional discharge process from ICU to medical wards and then home. Although there are differences between the CCRU and a traditional ICU, it seems reasonable to extrapolate from previous studies that direct discharge from the CCRU would translate to similar outcomes for patients.

This study aimed to investigate the outcomes of patients who were directly discharged from the CCRU. Specifically, we looked at the number of repeat emergency department (ED) visits or hospital readmissions and mortality within 6 months of discharge. Secondary objectives included the number of ED visits or hospital readmissions within 12 months and greater than 12 months following CCRU discharge. Additionally, we assessed patient demographics and clinical characteristics within this population that were more likely to be associated with higher rates of ED or hospital readmission. This study's findings would have more implications for the clinical practice for other resuscitation units regarding discharging their patients.

## 2. Methods

### 2.1. Study Design and Patient Selection

This was a retrospective study involving all adult nontrauma patients who were admitted to the CCRU between January 01, 2017, and December 2020. Any patients who were discharged directly from the CCRU to any destination, including hospice facilities, would be eligible. Patients who died while being in the CCRU or being transferred to inpatient palliative care service were excluded. Our study was exempted by our Institutional Review Board for formal consent, due to its retrospective nature (HP-00084554).

The CCRU is a 6-bed intensive care unit (ICU)-based resuscitation unit that was designed to expedite the transfer of patients with critical illnesses or time-sensitive diseases when there are no available beds at an appropriate traditional ICU at our institution. The CCRU is staffed 24 hours per day by an attending physician dually certified in emergency medicine and critical care medicine. Additionally, the CCRU is also staffed with an Advanced Practice Practitioner with critical care experience. To meet the ratio of 2 patients to 1 nurse, the unit is also staffed by 3 nurses who have at least 2 years of ICU experience. In addition to the 3 nurses, the charge nurse can also help to take care of 1 to 2 patients, which would enable certain critically ill patients to be cared for by more than 2 bedside nurses during resuscitation or certain life-saving procedures.

When the clinicians at the referring facilities contact our University of Maryland Medical Center's Access Center, which handles all interfacility transfers, the team at the Access Center will connect the referring clinicians with the on-call specialty physician and the appropriate ICU physician, if necessary. If the patient would need immediate intervention or an ICU level of care and there is no available ICU bed, the CCRU physician will be contacted for bed request. During this phone transfer request, the CCRU physician would discuss the plan of care for the patient from the referring hospital until arrival at the CCRU. When the patients arrive at the CCRU, the specialty consulting teams will assess the patients and provide any definitive plan of care, in conjunction with the CCRU team. The patients will receive the resuscitation or necessary interventions in the CCRU until there are appropriate beds at another inpatient unit. These resuscitation efforts include advanced methods such as venovenous or venoarterial extracorporeal membrane oxygenation (ECMO), intraaortic balloon pump, external ventricular drain (EVD) for patients with intracerebral hemorrhage, and continuous electroencephalogram (EEG), in addition to basic methods such as invasive mechanical ventilation, continuous renal replacement therapy, vasopressors, or antihypertensive infusions.

### 2.2. Outcome Measure

Our primary outcome was the number of ED visits or hospital admissions within 6 months of discharge from the CCRU. We did not consider any visits to an outpatient clinic or telephone calls as unplanned visits. Thus, these visits were not counted as the number of ED visits or hospital admissions. Any ED visits that lead to hospital admission only counted as 1 encounter. The secondary outcomes were the number of ED visits or hospital admissions during the 12 months after CCRU discharge, greater than 12 months from discharge.

### 2.3. Data Collection and Management

Patients' demographic and clinical data were collected from our institutional electronic medical records (Epic, Verona, Wisconsin, USA) according to the best practices as suggested [9]. Collected data included patients' demographic information (age, gender, past medical history), clinical information upon arrival, and leaving the CCRU (components of the Sequential Organ Failure Assessment [SOFA] score, serum lactate levels, hemoglobin levels, types of interventions), and disposition from the CCRU. Missing laboratory data was imputed as normal.

For post-CCRU discharge outcome, the investigators examined 2 sources of electronic medical records. We first examined the electronic medical records in Epic ecosystem that was available to our institution. To ensure that we did not miss the outcome data in other electronic medical records, we also examined our patients' records within our statewide health information exchange (https://www.crisphealth.org). This health information exchange contains all electronic medical records among hospitals within the regions, regardless of their systems of electronic medical records.

Research team members were not blinded to the study hypothesis when they were trained by the Principal Investigator for data collection. The junior investigators were first trained to identify and enter data into a standardized Excel spreadsheet (Microsoft Corp., Redmond, Washington, USA), using blocks of 10 patients' charts. Training is complete once their data's accuracy was ≥90% matching with a senior investigator's data. Any discrepancies were adjudicated among the investigators and the Principal Investigator. Missing components for the SOFA score or laboratory values were imputed with normal values.

### 2.4. Statistical Analysis

Due to the retrospective and descriptive nature of this study, we did not calculate the sample size for the study.

Prior to analyzing data, we examined the histograms of continuous data for their patterns of distributions. Normally-distributed data was expressed as mean (± standard deviation (SD)), and nonparametric data was expressed as median (interquartile range (IQR)). Continuous data for patients at CCRU admission and discharge was analyzed via paired *T*-test or Mann–Whitney *U* test as appropriate. Categorical data was expressed as percentage and analyzed with the Chi square test.

To examine the association between patients' demographic and clinical data and the number of ED or hospital visits, multivariable ordinal logistic regressions were performed for each of the study period (within 6 months of discharge, within 12 months of discharge, and >12 months of discharge. The order of ED visits for the ordinal logistic regression was ranked from 0 (no ED visit or hospital admission) to 1 (1 visit during a specified study period), to 2 (2 visits), and to 3 (>3 visits during the study period). We identified these variables a priori according to our clinical practice as all of these variables are part of our resuscitation and evaluation of patients in the CCRU. The list of independent variables was included as Table 1. The results were expressed as coefficients and 95% confidence intervals (95% CI) of the coefficient. Any positive coefficients would make 0 visits most likely while negative coefficients would make 3+ visits most likely.

All statistical analyses were performed with Minitab version 19 (https://www.minitab.com; State College, Pennsylvania, USA). All statistical tests with *P* value <0.05 were considered statistically significant.

## 3. Results

### 3.1. Patient Demographics and Hospital Outcomes

There was a total of 6969 patients who were admitted to the CCRU during the study period. We identified and analyzed records of 145 (0.02%) patients. The mean (± standard deviation) for age was 55.6 (19.2) years and 104 (71.7%) were male (Table 2). The median (interquartile range (IQR)) between discharge and chart follow-up was 4 [3.1–4.9] years. The most common admitting diagnosis was soft tissue infection (Table 2). The list of admitting diagnoses is in Table 3. The most common reason for discharge was “symptom improvement.”

Within 6 months of discharge, 60 (41.4%) of the patients presented to an ED or were admitted to the hospital. The number of patients increased to 79 (54.5%) and 114 (78.6) within 12 months and beyond 12 months, respectively. There were 7 patients who died within 6 months and they were discharged to hospice care (Table 2).

### 3.2. Interventions

There were 14 (9.7%) patients who required mechanical ventilation at admission to the CCRU (Table 4). The majority of the patients (100, 68.9%) would require 1 or more transfusions of continuous medications, while only 16 (11%) patients received transfusion of 1 blood type and 2 (1.4%) received transfusions of 2 or more blood products; patients did not have high acuity as the mean SOFA score was 1.6 (±2.0) and their mean serum lactate levels were at the upper limit of normal (Table 5).

### 3.3. Predictors for ED Visits or Hospital Readmission

The multivariable ordinal logistic regressions showed that requiring mechanical ventilation at admission to the CCRU (coefficient −2.23, 95% CI 0.01–0.87, *P* = 0.036) was associated with the highest risk for greater than 3 ED visits or hospital admission within 6 months of discharge (Table 6). For risk of +3 ED revisits or hospital admission within 12 months, patients with mechanical ventilation (coeff. −2.07, 95% CI 0.02–0.93, *P*=0.043) and soft tissue infection (coeff. −2.05, 95% CI 0.03–0.47, *P*=0.002) were associated with high risk.

Patients with soft tissue infection (coeff. −1.97, 95% CI 0.03–0.58, *P*=0.006) were associated with high risk for +3 ED visits or hospital admissions in 12 months or more since CCRU discharge (Table 6).

## 4. Discussion

Limited data exist looking at outcomes of patients discharged from the ICU. Our study is unique in that it specifically looks at recurrent ED visits or hospitalizations in patients discharged from a tertiary care referral center resuscitation unit, whose patient population is often critically ill on admission. Our study indicated that among the small number of patients who were discharged directly from the CCRU, there was a low number of unwanted outcomes as only patients who were discharged to hospice or palliative care (family decision) died within 6 months from discharge. Our study also identified a few factors that could have been associated with unplanned ED visits or hospital admissions, which would potentially help clinicians to devise the plan of care.

The rate of ED visits or hospital readmission from this study was significantly different from the rates of direct discharge from traditional ICUs as reported by Martin et al. [5]. The population-based study by Martin et al. reported a rate of 23.7% of ED visits or hospital admission within 30 days of ICU discharge; on the other hand, the rate was 41% at 6 months for our population, despite our patients having lower SOFA score. There were likely multifactorial reasons for these differences. First, this study performed a longer follow-up, up to 6 months after discharge. Furthermore, previous studies such as those by Martin et al. and Stelfox et al. reported a number of patients who were admitted for less chronic disease such as drug overdose. On the other hand, our patient population was mostly patients with diseases with high morbidity such as aortic diseases, ischemic stroke, or soft tissue infection. These patients are potentially at high risk of exacerbation of their chronic conditions. On the other hand, many patients who underwent surgical interventions for necrotizing soft tissue infection would need readmission, for skin graft procedures or due to frequent episodes of reinfected wounds.

Another difference between the CCRU patient population and other studies reporting direct discharge from traditional ICUs would be the rates of discrepancy in diagnosis at transfer request and at the CCRU. Approximately 8% of the CCRU patients were discharged home because their diagnoses from the referring facilities did not match the diagnoses after further evaluation at the CCRU and thus did not warrant further hospitalization. When the patient arrives at the CCRU, the specialty consulting team that accepts the patient for transfer will review any available imaging studies, will evaluate patients, and then would obtain additional imaging studies for more definitive decision. When additional information at the CCRU suggests a different diagnosis that does not warrant further hospitalization, then patients can be discharged home. An anecdotal clinical example for these conditions includes patients who were transferred urgently to the CCRU for management of suspected type A aortic dissection that was seen on either computer tomography (CT) imaging without contrast or for pulmonary embolism (PE) protocol, while the patient also has normal blood pressure. Upon arrival at the CCRU, the patient would undergo a specialized electrocardiogram (ECG)-gated CT with angiogram, which would confirm nor refute the presence of the aortic dissection with high sensitivity and specificity [10]. If there was no dissection from the ECG-gated CT angiogram, the patient would be discharged. The findings of diagnostic discrepancies are unique for any resuscitation unit in that this further emphasizes the importance of close cooperation between the clinicians from the referring facilities, the specialty consulting service, and the resuscitation unit to ensure the proper and necessary transfer of patients to a tertiary care facility, especially when resources are limited.

Our study is important because it looks at the feasibility and safety of discharging patients from a high-acuity resuscitation unit. In today's saturated hospital systems, often operating near 100% capacity, there is an emphasis on ICU cost reduction and rapidity of hospital discharges, including from the ICU, when appropriate [11]. Findings from this study suggested that patients who were discharged directly from the CCRU were associated with improving physiological parameters, which was consistent with previous observations about the CCRU's effectiveness in lowering SOFA score and patients' improved outcome [12]. Thus, it is probably safe to discharge patients, even from a resuscitation unit, once the patients' symptoms or physiological parameters improved. This possibility can be applied to patients at any referring facility, after appropriate testing and management. Further studies are necessary to investigate the safety and impact of this strategy on patients' satisfaction, outcome, and resource utilization.

This study was also different from previous studies involving ICU discharged patients [5, 13–15] that this study included patient-level information that might have been associated with their likelihood of unplanned ED visits or hospital admission. While certain patient-level variables such as mean arterial pressure at arrival, requiring mechanical ventilation during CCRU stay, hemoglobin levels were intuitive, the higher number of telehealth visits were associated with higher number of patients' ED visits or hospital admission from 6 months to >2 years after CCRU discharge was counterintuitive. Telemedicine has been shown to be comparable with traditional types of care among patients with chronic disease [16]. However, it was likely that patients with chronic disease would tend to adhere to follow-up more frequently and they were likely to require more ED visits or hospital admission. We could not exclude the reverse causality, in which patients with frequent telehealth visits are more likely to be prompted to present to ED by the telehealth clinicians if patients' conditions deteriorate. Further studies are necessary to confirm our observations. Additionally, many patients who underwent surgical interventions for necrotizing soft tissue infection would need readmission, for skin graft procedures or due to reinfected wounds.

### 4.1. Limitations

Our study has several limitations. Although we searched both Epic ecosystem of electronic health records and the regional health information exchange, there are still possibilities that we would have missed patients' records. The SOFA scores for the patients who were discharged were relatively low because of the diverse and heterogenous patient population. The SOFA score would be low for patients with acute aortic disease and ischemic stroke which accounted for a large percentage of the discharged patients. Furthermore, we would not be able to detect ED visits if a patient was discharged to a skilled nursing facility and died before visiting an ED, as records from nursing home facility are not available at the statewide health information exchange. We also did not assess whether patients' readmission to the hospital was to ICU level, because distinguishing the reasons and the level of these hospital admissions were beyond the scope of our retrospective study and would warrant further studies. Similarly, our pilot study did not compare the postdischarge outcomes between the directly discharged patients versus others who were not discharged directly, as these data were not available to us. This comparison will also allow clinicians to have a better understanding of this patient population.

## 5. Conclusion

Direct discharge from the CCRU was uncommon but more than 50% of patients did not represent to an ED or require hospital admission within the first 6 months of discharge. A few clinical variables during patients' CCRU stay, such as requiring mechanical ventilation and having soft tissue infection were correlated with higher numbers of unplanned hospital revisits following discharge. Further research is needed to validate these findings.

## Figures and Tables

**Table 1:** Variables for ordinal logistic regressions. All variables were included in the models; we only reported statistically significant variables in the article.

*(1) Continuous variables*
Age (years)
SOFA score at CCRU arrival
SOFA score at CCRU discharge
‘MAP at CCRU arrival
‘MAP at CCRU discharge
WBC at CCRU arrival
WBC at CCRU discharge
‘Hemoglobin at CCRU arrival
‘Hemoglobin at CCRU discharge
Lactate at CCRU arrival
Lactate at CCRU discharge
Troponin at CCRU arrival
Troponin at CCRU discharge
Total number of infusions
Total number of transfusions
Total number of telemedicine visits

*(2) Categorical variables*
Gender, female
Race
Past medical history
Hypertension
Diabetes
Liver disease
Kidney disease
Any heart disease
Requiring central line
Requiring invasive mechanical ventilation
Diagnoses

CCRU, critical care resuscitation unit; MAP, mean arterial pressure; SOFA, sequential organ failure assessment; WBC, white blood cell count.

**Table 2:** Demographic information of patients who were discharged directly from the critical care resuscitation unit (CCRU).

Variables	All patients (*n* = 145)	95% CI for %
Age in years, mean (SD)	55.6 (19.2)	N/A

*Gender, N (%)*		
Male	104 (71.7)	(63.7, 78.9)
Female	41 (28.3)	(21.1, 36.3)

*Race, N (%)*		
Black	58 (40.0)	(32.0, 48.5)
White	84 (57.9)	(49.5, 66.1)
Hispanic	2 (1.4)	(0.2, 4.9)
Asian	1 (0.7)	(0.002, 3.8)

*Past medical history, N (%)*		
Hypertension	69 (47.6)	(39.2, 56.0)
Diabetes mellitus	27 (18.6)	(12.6, 25.9)
Coronary artery disease	15 (10.3)	(5.9, 16.5)
Congestive heart failure	11 (7.6)	(3.8, 13.2)
Hepatitis	16 (11.0)	(6.4, 17.3)
Cirrhosis	2 (1.4)	(0.2, 4.9)
Chronic kidney disease	9 (6.2)	(2.9, 11.5)
End stage renal disease	2 (1.4)	(0.2, 4.9)

*Length of stay and follow-up time, median (IQR)*		
CCRU length of stay in hours	24.3 (11.3, 41.2)	N/A
Time from discharge to last follow-up in years	4.0 (3.1, 4.9)	N/A

*Reason for discharge, N (%)*		
Against medical advice	5 (4.5)	(1.1, 7.9)
Discrepancies between diagnoses	12 (8.3)	(4.3, 14.0)
Family decision	8 (5.5)	(2.4, 10.6)
Symptoms improved/no longer required intervention	116 (80.0)	(72.6, 86.2)
Others	4 (2.8)	(0.8, 6.9)

*Patients that returned to the hospital, N (%)*		
0–6 months	60 (41.4)	(33.3, 49.8)
0–12 months	79 (54.5)	(46.0, 62.8)
Greater than 12 months	114 (78.6)	(71.0, 85.0)

*Average total hospital visits, mean (SD)*		
0–6 months	1.1 (2.1)	N/A
0–12 months	1.7 (2.8)	N/A
Greater than 12 months	4.9 (7.8)	N/A

*Patients with telehealth visit, N (%)*		
0–6 months	87 (60.0)	(51.5, 68.0)
0–12 months	91 (62.8)	(54.3, 70.6)
Greater than 12 months	99 (68.3)	(60.0, 75.7)

*Average total telehealth visits, mean (SD)*		
0–6 months	2.9 (4.9)	N/A
0–12 months	4.1 (6.9)	N/A
Greater than 12 months	9.4 (15.8)	N/A

*Mortality, N (%)*		
Mortality within 6 months of discharge	7 (4.8)	(2.0, 9.7)

*Most common admitting diagnoses, N (%) * ^1^		
Soft tissue infection	24 (16.6)	(10.9, 23.6)
Acute aortic syndrome	21 (14.5)	(9.2, 21.3)
Stroke	16 (11.0)	(6.4, 17.3)
Trauma	14 (9.7)	(5.4, 15.7)
Hemorrhage	7 (4.8)	(2.0, 9.7)
Respiratory failure	6 (4.1)	(1.5, 8.8)

*Most common diagnoses at discharge, N (%) * ^1^		
Soft tissue infection	24 (16.6)	(10.9, 23.6)
Acute aortic syndrome	21 (14.5)	(9.2, 21.3)
Stroke	16 (11.0)	(6.4, 17.3)
Trauma	14 (9.7)	(5.4, 15.7)
Hemorrhage	7 (4.8)	(2.0, 9.7)
Respiratory failure	7 (4.8)	(2.0, 9.7)

^1^Only the top 5 common diagnoses were reported here. CCRU, critical care resuscitation unit, IQR, interquartile range; SD, standard deviation.

**Table 3:** Diagnoses at admission and discharge of patients who were discharged directly from the critical care resuscitation unit (CCRU).

Diagnosis	Admission, *N* (%)	Discharge, *N* (%)
Acute liver injury	1 (0.7)	0 (0)
Acute aortic syndrome	21 (14.5)	21 (14.5)
Altered mental status	2 (1.4)	2 (1.4)
Aortic dissection	1 (0.7)	0 (0)
Appendicitis	1 (0.7)	1 (0.7)
Arterial occlusion	3 (2.1)	3 (2.1)
Brain mass	2 (1.4)	2 (1.4)
Coronary artery disease	4 (2.8)	4 (2.8)
Foreign body ingestion	5 (3.5)	5 (3.5)
Headache	2 (1.4)	2 (1.4)
Hemorrhage	7 (4.8)	7 (4.8)
Intraabdominal abscess	1 (0.7)	1 (0.7)
Intracerebral hemorrhage	2 (1.4)	1 (0.7)
Intracranial hemorrhage	5 (3.5)	5 (3.5)
Intraparenchymal hemorrhage	1 (0.7)	1 (0.7)
Ischemic bowel	1 (0.7)	1 (0.7)
Mesenteric ischemia	2 (1.4)	2 (1.4)
Pancreatitis	1 (0.7)	1 (0.7)
Pulmonary artery dissection	1 (0.7)	1 (0.7)
Respiratory failure	7 (4.8)	7 (4.8)
Retropharyngeal injury	1 (0.7)	0 (0)
Seizure	5 (3.5)	5 (3.5)
Sepsis	2 (1.4)	1 (0.7)
Sigmoid volvulus	1 (0.7)	1 (0.7)
Soft tissue infection	24 (16.6)	24 (16.6)
Stroke	16 (11.0)	16 (11.0)
Subarachnoid hemorrhage	4 (2.8)	4 (2.8)
Subdural hematoma	1 (0.7)	1 (0.7)
Syncope	1 (0.7)	1 (0.7)
Traumatic brain injury	1 (0.7)	1 (0.7)
Toxicologic emergency	1 (0.7)	1 (0.7)
Trauma	14 (9.7)	14 (9.7)
Urticaria	1 (0.7)	1 (0.7)
Intracranial aneurism	1 (0.7)	1 (0.7)
Renal failure	2 (1.4)	2 (1.4)
Acute renal failure	0 (0)	1 (0.7)
Infected aortic graft	0 (0)	1 (0.7)
Intracranial bleed	0 (0)	1 (0.7)
Pharyngeal laceration	0 (0)	1 (0.7)
Ascending aortic aneurysm	0 (0)	1 (0.7)

**Table 4:** Clinical interventions provided in the critical care resuscitation unit (CCRU).

Patients receiving clinical intervention, *N* (%)	All patients (*n* = 145)	95% CI
Central venous catheter	3 (2.1)	(0.4, 5.9)
Mechanical ventilation	14 (9.7)	(5.4, 15.7)

*Infusions, N (%)*		
0 infusions	45 (31.0)	(23.6, 39.2)
1 infusion	73 (50.3)	(41.9, 58.7)
2+ infusions	27 (18.6)	(12.6, 25.9)

*Blood transfusions, N (%)*		
0 transfusions	127 (87.6)	(81.1, 92.5)
1 type of blood products	16 (11.0)	(6.4, 17.3)
2+ types of blood products	2 (1.4)	(0.2, 4.9)

**Table 5:** Comparison of laboratory values at the time of admission and discharge of patients who were discharged directly from the critical care resuscitation unit (CCRU).

Variables, mean (SD)	Admission	Discharge	*P* value
Mean arterial pressure (mmHg)	99.8 (17.1)	95.0 (14.9)	0.002
White blood cell (count/*μ*L)	9.8 (4.8)	8.4 (3.5)	<0.001
Hemoglobin (g/dL)	12.4 (2.4)	12.5 (2.0)	0.568
Lactate (mg/dL)	1.9 (1.6)	1.2 (0.5)	<0.001
Troponin (ng/ml)	0.03 (0.08)	0.03 (0.11)	0.878
SOFA score	1.6 (2.0)	0.9 (1.1)	<0.001
Change in SOFA	N/A	−0.8 (1.7)	N/A

g/dL, gram per deciliter; mg/dL, milligram per deciliter; mmHg, millimeter of mercury; ng/ml, nanogram per milliliter; SD, standard deviation; SOFA, sequential organ failure assessment; *μ*L, microliter.

**Table 6:** Multivariable ordinal regressions for association of demographic and clinical information with the number of emergency department visits or hospital admission within 6 months, within 12 months, and greater than 12 months of the critical care resuscitation unit (CCRU) discharge.

Predictor	Coeff.	*P* value	Odds ratio	95% CI	
				Lower	Upper
*ED visits or hospital admission within 6 months of CCRU discharge*					
MAP arrival	−0.034	0.014	0.97	0.94	0.99
Number of infusions	−0.744	0.039	0.47	0.23	0.96
Number of tele visits 0–6	−0.18	0.001	0.83	0.76	0.92
History of liver disease	−1.40	0.015	0.24	0.08	0.76
History of mechanical ventilation	−2.23	0.036	0.11	0.01	0.87
Soft tissue infection	−1.79	0.009	0.17	0.04	0.64

*ED visits or hospital admission within 12 months of CCRU discharge*					
MAP arrival	−0.031	0.018	0.97	0.94	0.99
Number of tele visits 0–12	−0.15	0.001	0.86	0.79	0.93
History of liver disease	−1.48	0.013	0.23	0.07	0.73
History of kidney disease	−2.03	0.013	0.13	0.03	0.66
Requiring mechanical ventilation	−2.07	0.043	0.13	0.02	0.93
Soft tissue infection	−2.05	0.002	0.13	0.03	0.47
Hemoglobin at discharge	0.32	0.018	1.38	1.06	1.81

*ED visits or hospital admission after more than 12 months from CCRU discharge*					
Number of tele visits	−0.093	0.001	0.91	0.87	0.96
Soft tissue infection	−1.97	0.006	0.14	0.03	0.58
Hemoglobin at admission	−0.31	0.017	0.73	0.56	0.95
Hemoglobin at discharge	0.42	0.004	1.54	1.15	2.06

Coeff., coefficient; ED, emergency medicine; g/dL, gram per deciliter; MAP, mean arterial pressure. The order was ranked as 0 (0 visits), 1 (1 visit), 2 (2 visits), and 3 (3 or more visits). Positive coefficients make 0 visits most likely while negative coefficients make 3+ visits most likely. Only statistically significant independent variables are reported in this table.

## Data Availability

All of the data are private and not available for third party use or view.
